# A Systematic Review of Lipid Management in Secondary Prevention and Comparison of International Lipid Management Pathways

**DOI:** 10.7759/cureus.35463

**Published:** 2023-02-25

**Authors:** Zahid Khan, Amresh Gul, Yousif Yousif, Animesh Gupta

**Affiliations:** 1 Acute Medicine, Mid and South Essex NHS Foundation Trust, Southend on Sea, GBR; 2 Cardiology, Bart’s Heart UK, London, GBR; 3 Cardiology and General Medicine, Barking, Havering and Redbridge University Hospitals NHS Trust, London, GBR; 4 Cardiology, Royal Free Hospital, London, GBR; 5 General Practice, Lifeline Hospital, Salalah, OMN; 6 Internal Medicine, Barking, Havering and Redbridge University Hospitals NHS Trust, London, GBR; 7 Acute Internal Medicine, Southend University Hospital NHS Trust, Southend on Sea, GBR; 8 Acute Internal Medicine/Intensive Care, Barking, Havering and Redbridge University Hospitals NHS Trust, London, GBR

**Keywords:** cardiac risk factors and prevention, cardiac mortality, primary care medicine, phase-i cardiac rehabilitation, myocardial infarction, pcsk9 inhibitors vs statins, risk factors cardiovascular diseases, cardiovascular prevention, : acute coronary syndrome, dyslipidaemia

## Abstract

Acute coronary syndrome remains a major cause of morbidity and mortality despite significant improvements in its prevention and management. Lipid management and other risk factors such as hypertension, diabetes, obesity, smoking and sedentary lifestyle stratification is the key to minimising this risk. Lipid management is an important part of secondary prevention and patients are historically undertreated after post-acute coronary syndrome. We performed a narrative review on observational studies on lipid management pathways post ACS on PubMed, Google Scholar, Journal Storage and ScienceDirect and excluded case reports, case series and randomized controlled trials. Our review showed that most patients following acute coronary syndrome receive suboptimal treatment for hypercholesterolemia. The role of statin in reducing future cardiac events risk is undisputable, however, statin intolerance remains a major concern. There is substantial variation in the management of lipids in patients following an acute cardiac event and patients were followed up in primary care in some countries and secondary care in others. The mortality risk is significantly high in patients with second or recurrent cardiac events and future cardiac events are associated with higher morbidity and mortality risk. There is significant variation in lipid management pathways in patients who suffer from cardiac events across the globe and lipid therapy optimization remains suboptimal in these patients, putting them at future risk of cardiovascular events. It is therefore imperative to optimally manage dyslipidemia in these patients in order to minimize the risk of subsequent cardiac events. Cardiac rehabilitation programs might be a way forward to incorporate lipid management for patients discharged from the hospital after having acute coronary events for lipid therapy optimization.

## Introduction and background

The in-hospital mortality rate of myocardial infarction (MI) has reduced significantly over the past two to three decades due to improved treatment strategies [1,2]. Despite recent advances in management strategies, acute coronary syndrome (ACS) remains a major cause of global mortality and morbidity, and patients with ACS are at very high risk for future adverse cardiovascular events [3]. Although most patients with ACS are given high-intensity statins for secondary prevention, a significant number of patients still remain undertreated and as a result, the therapeutic goals are not met. There is slight variation in the international guidelines when it comes to lipid management in patients post-ACS and calculating individual patient risk is important in decision-making. The target lower-density lipoprotein cholesterol (LDL-C) levels vary for patients depending on their risk factors and whether they had single or multiple cardiac events. Research has shown that high concentrations of LDL, intermediate-density lipoprotein (IDL), very low-density lipoprotein (VLDL) and low concentrations of high-density lipoprotein (HDL) are commonly associated with a higher risk of ischaemic heart disease (IHD) [2-4].

The European Society of Cardiology (ESC) 2021 guidelines recommend a target reduction of 50% reduction in LDL-C levels and physicians should aim for a value < 1.4 mmol/L (55mg/dL) in individuals with recent MI [4]. The ESC guidelines recommend a repeat lipid profile check in patients with ACS in four to six weeks' time to optimize their lipid therapy. The National Institute for Health and Care Excellence (NICE) guidelines on the other hand recommend a 40% reduction in non-high-density lipoprotein cholesterol (non-HDL-C) in patients with ACS and recommend a repeat lipid profile check in three months' time after initiation of the statin therapy. The NICE guidelines recommend initiating statin therapy in both primary and secondary prevention to manage modifiable risk factors and high-dose statins should be prescribed to patients with ACS [5,6]. The aim of this systematic review is to compare the different international lipid management pathways and highlight the potential differences between them. In addition, this review will identify factors that could be contributing to non-adherence to the guidelines on lipid therapy optimization. We will also briefly discuss the literature on integrating lipid therapy optimization into cardiac rehabilitation.

## Review

Methodology

**Figure 1:**
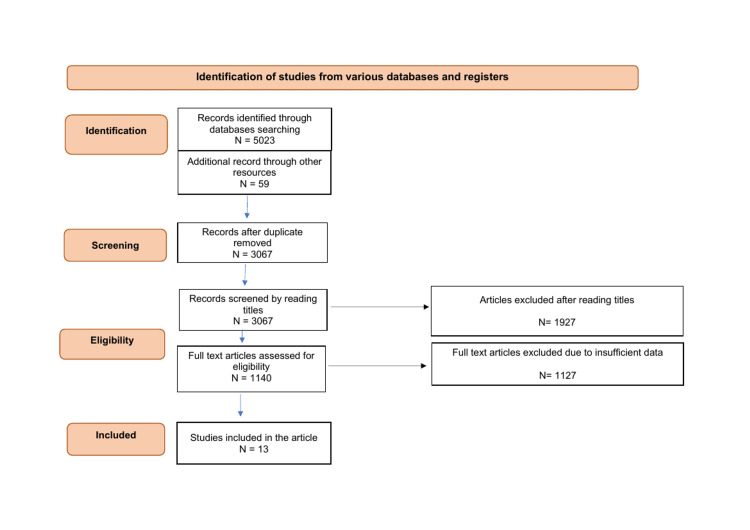
Preferred Reporting Items for Systematic Reviews and Meta-Analyses (PRISMA) Chart for the narrative review.

We performed a systematic review of the available literature on lipid pathways by searching for literature on various search engines including PubMed, Google Scholar, Journal Storage (JSTOR) and ScienceDirect. We reviewed a total of 5082 articles published between 2020 and 2023 and a total of 5023 articles using keywords such as "Lipid management and acute coronary syndrome, lipid management and ACS, lipid management and myocardial infarction, cardiac rehabilitation and lipid management, acute coronary syndrome and lipid-lowering therapy, lipid-lowering medications and acute coronary syndrome, mixed dyslipidaemia and ACS, combination therapy for lipid management and ACS, international lipid management pathway in ACS, lipid pathway and ACS". We arrived at a total of 13 articles after removing duplicate articles, articles with study participants < 18 years, articles that were not freely accessible, articles not written in the English language, case series, case reports and randomized controlled trials (RCTs). The RCTs were excluded as the aim of this review was to assess and identify issues with the various lipid pathways that can hinder medicine optimization in patients with IHD. The Preferred Reporting Items for Systematic Reviews and Meta-Analyses (PRISMA) chart summarizing the literature search is presented below (Figure 1).

Inclusion Criteria

Studies on patients' lipid management post-ACS follow-up in primary or secondary care and studies with participants aged> 18 years were included in this study.

Exclusion Criteria

Studies with participants < 18 years old, case reports, case series, randomized controlled trials, and cohort studies with no follow-up were excluded.

Results and comparison of the various international lipid management initiatives discussion

Patients with high LDL-C and low HDL-C are at high risk of acute cardiac events. Both genetic and non-genetic factors predispose individuals to an increased risk of atherosclerotic cardiovascular disease (ASCVD). Dyslipidaemia is one of the most important contributors to this risk due to its ability to enhance atherosclerosis. Patients who suffer from ACS usually do not receive the optimal therapy to reduce their future risk due to a lack of efficient follow-up to optimize lipid-lowering therapies. The aim of this review is limited to comparing and discussing various international lipid pathways in patients following ACS despite the availability of adequate pharmacological therapies which puts them at increased risk of future cardiac events due to atherosclerosis. There is also variation in the guidelines regarding the recommended target LDL-C levels for patients with ACS, however, despite that, most patients fail to achieve the recommended LDL-C levels following myocardial infarction. The European Society of Cardiology guidelines recommend a significantly lower LDL level <1.4 mmol/L in patients post ACS whereas current SIGN and NICE guidelines recommend a reduction in LDL-C by 40% or level <1.8 mmol/L for moderate to high risk and 1.4 mmol/L in very high-risk patients. The "EUROASPIRE V" survey demonstrated that only 30% of patients achieved low-density lipid cholesterol (LDL-C) levels < 1.8 mmol/L at 12 months following their discharge from the hospital after suffering acute myocardial infarction [5]. Similarly, the "ACS EuroPath" survey showed a considerable lack of compliance among physicians with guidelines for managing lipids in patients post-ACS due to various reasons [7]. This led to the initiative of Europe-wide initiatives to improve lipid management in patients with ACS during hospital admission and after discharge.

**Figure 2:**
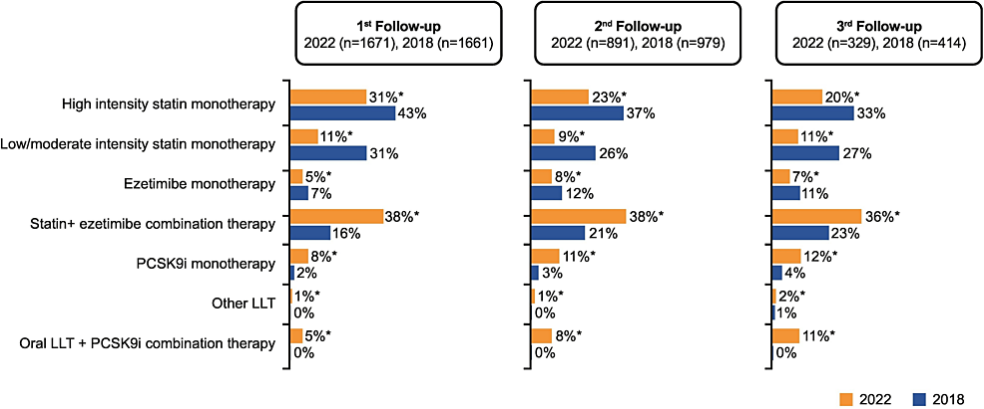
Pharmacological approach at 1st, 2nd and 3rd follow-ups during chronic phase in the ACS EuroPath IV project ACS - Acute coronary syndrome [7]

**Figure 3:**
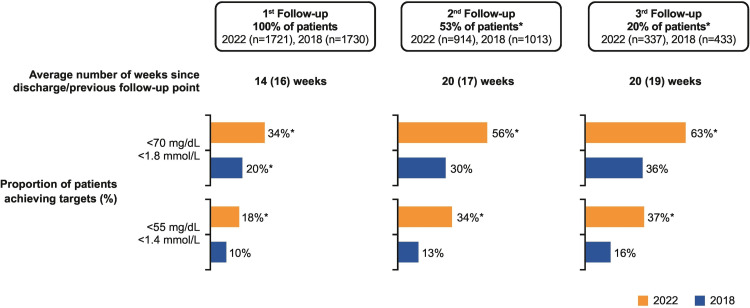
Achievement of LDL-C goals at 1st, 2nd and 3rd follow-up in the ACS EuroPath IV project ACS - Acute coronary syndrome [7]

The ACS EuroPath study involving 555 cardiologists compared the lipid pathways from seven European countries in patients following ACS. This study demonstrated that 87% of patients (546/626) did not achieve the recommended lipid level on the second follow up and 75% of patients (578/774) were not commenced on additional lipid-lowering therapy on the first follow-up despite not achieving the target lipid levels. Around 64% of patients with ACS were not followed up within six weeks according to this study and there was significant variation in the lipid pathways amongst these countries. For example, the lipid management pathway for ACS patients in the Netherlands was based on follow-up of patients with LDL-C > 70 mg/dL by cardiologists within three months and intensified lipid-lowering therapy if the target was not achieved. A combination therapy of high-dose statin and ezetimibe was recommended for statin-naive patients with LDL-C > 100 mg/dL. The pathway in Spain was based on virtual follow-up of patients on a six-weekly basis till patients achieved the target LDL-C levels. The pathway in Italy was different as patients were referred to primary care for follow-up and to have repeat lipid profiles in a month's time; General practitioners would refer patients back to cardiologists if LDL-C levels were not achieved. Patients were followed by cardiac rehabilitation nurses in Sweden who were responsible for optimizing lipid-lowering therapy if target levels were not met [3]. This led to the initiation of the "ACS EuroPath II" project that led to the development of self-assessment tools for these high-risk patients during the acute and follow-up phases to optimize their lipid-lowering therapy based on the ESC 2019 guidelines. This project was based on four phases including the acute phase, discharge phase, follow-up phase and referral pathways planning for patients who did not achieve the target LDL-C levels [6,7]. The aim of this project was to assess the effectiveness of local pathways to manage high lipid levels in patients with ACS and to achieve the target levels by optimizing medical therapy. Following this, the "ACS EuroPath IV" project in 2022 compared 2650 patients from six European countries treated for ACS between March - June 2022 and compared this data with 2650 patients in ACS EuroPath I survey from 2018. This study demonstrated that lipid testing in patients with ACS had improved and 90% of patients had their lipid profiles checked within a mean of 1.4 days compared to 1.7 days in 2018. This project also showed an increasing tendency to test patients for non-HDL-C, lipoprotein (a) (Lpa) and apolipoprotein B (ApoB) compared to 2018. There was variation in the prescription of lipid-lowering therapy and 34% of patients received a combination therapy of statin and ezetimibe vs 13% in 2018. Similarly, 90% of patients were discharged on lipid-lowering therapy on discharge from the hospital in the "ACS EuroPath IV" project. A higher percentage of patients achieved lower LDL-C levels in 2022 in a shorter duration; 34% of patients vs 20% achieved a target level of LDL-C < 70 mg/dL and 18% vs 10% achieved a level < 55 mg/dL in 2022 vs 2018 project. The duration to achieve these targets was 14 weeks in 2022 vs 16 weeks in 2018 [8]. Another important finding in this project was the fact that 44% of patients were receiving high-dose statin vs 59% in 2018 and only 12% vs 25% of patients were receiving low/moderate intensity statin monotherapy in comparison to 2018. A total of 56% vs 30% of patients achieved target LDL-C levels < 70 mg/dL and 34% vs 13% achieved a target level of < 55 mg/dL on their second follow-up in the 2022 project compared to 2018. The duration to achieve this target was 20 vs 17 weeks following the first follow-up in 2022 compared to 2018. By the 3rd follow-up, 63% vs 36% of patients achieved LDL-C levels < 70 mg/dL and 37% vs 16% achieved LDL-C levels < 55 mg/dL in 20 weeks vs. 19 weeks in the "ACS EuroPath IV" project vs ACS EuroPath I project. An encouraging aspect of both projects is that > 90% of patients received lipid-lowering therapy during admission. Lpa was tested in 30% vs 15%; ApoB was tested in 7% and 4% in 2022 and 2018 respectively. About 90% of patients were discharged with LLT in 2022 vs 93% in 2018 and 59% vs 44% of patients on high-intensity statin therapy in 2018 vs 2022 however the percentage of patients on statin and ezetimibe therapy was 34% vs 13% in 2022 vs 2018. None of the patients received PCSK9 inhibitor therapy in 2018 whereas 2% of patients received this monotherapy in the 2022 project. Despite this progress, lipid management in patients with ACS remains sub-optimal. The comparison between the two projects is shown in Figures 2-3.

The PENELOPE study from the Netherlands enrolled high-risk ACS patients six weeks following ACS between 1-1-2019 and 31-08-2020 and it demonstrated that 99% high-risk ACS patients based on ESC guidelines definition achieved the target goal of LDL < 1.8 mmol/L in 12 weeks and only 10% patients required PCSK9 inhibitors in this group [3]. It recommended stepwise intensification of lipid-lowering therapy (LLT) if target LDL-C was not achieved. The study established an ESC guideline-endorsed algorithm for high-risk patients with a three-month follow-up visit following ACS and it rendered a step-wise lipid modification therapy for patients with LDL >70mg/dl (>1.8 mmol/L). The POSTER study from Italy enrolled high-risk ACS patients who had a significant risk for FH and the main aim of this study was to improve the coordination between cardiologists and lipidologists in optimizing lipid-lowering therapy in these patients [5]. The Belgian consensus panel made up of cardiologists, endocrinologists, lipidologists, and cardio-geneticists drafted the Belgium Familial Hypercholesterolemia Strategy (BEL-FaHST protocol) in 2017 for identifying and managing patients with FH following ACS. This study categorized patients into two alert groups for screening purposes, with patients < 65 years old suffering from ACS placed in one group and patients with LDL-C > 190 mg/dL without statin therapy or 130 mg/dL on statin therapy placed in the second group [5]. Being a strict criterion, 90% of patients in this study met neither of these criteria and were discharged on high-intensity statins or combination therapy of statin and ezetimibe if already on statins. The “SWEDEHEART” is a nationwide registry study from Sweden for patients following AMI and it showed variability from 40%-75% in achieving the target LDL-C [6]. A major difference in this study compared to the other was that cardiac rehabilitation nurses were responsible for optimizing lipid-lowering therapy in patients who had not achieved target LDL-C levels [3,7]. A Danish population-based study based on 4377 patients with a mean age of 67.8 ± 13.6 showed that 89.4% of patients had LDL-C > 1.8 mmol/L at the time of ACS [9-11]. In comparison to other European countries, patients with ACS in Austria are generally followed up in the community for lipid-lowering therapy optimization and only patients with severely reduced left ventricular ejection fraction are followed up in a hospital setting [3,12]. The first follow-up for these patients usually happens several months after the initial cardiac event which predisposes them to additional risk in absence of optimal lipid management. Another major difference in this pathway is that the PCSK9 inhibitors can only be initiated by endocrinologists although the costs are reimbursable to patients and only 30% of patients achieve a target LDL-C level following acute coronary syndrome. Hence a new pathway was established by four cardiologists with lipid expertise to optimize lipid management in these patients. High-risk ACS patients on this new pathway with LDL-C>190 mg/dL or statin intolerance, patients with LDL-C>100 mg/dL if already established on statin therapy, Lp(a) > 150 nmol/L, age<45 years were discharged on high dose statins and ezetimibe and were followed up in outpatient rehab clinic within two months [13]. The Euroaspire V study approach was different as patients in this pathway were followed by cardiologists and general practitioners [14]. They initiated this pathway to ensure that patients commenced lipid-lowering therapy during hospital admissions and had virtual cardiology clinic follow-ups in six weeks’ time. This approach was different to the above-mentioned studies where patients were seen face to face by cardiologists, cardiac rehabilitation nurses or primary care physicians and patients who were followed up in the virtual clinic. Around 90% of patients in this study had repeat lipid profiles checked prior to the virtual clinic follow-up.

Cardiologists were able to commence alirocumab at the highest dose in patients with LDL-C >140 mg/dL in the PENLOPE study. The POSTER study demonstrated that 51% had LDL-C>70 (>1.8 mmol/L) and 5.1% of these patients had probably or definite FH a month following ACS. A total of 74.6%, 25.4% and 0.1% of patients were on statins only, combination therapy and PCSK9 inhibitors in this study and were referred to the lipid clinic [5]. The DLCN score was calculated for patients in the BEL-FaHST protocol study at three months following acute coronary syndrome and patients with LDL-C > 70 mg/dL despite being on lipid-lowering therapy for four weeks were commenced on combination therapy of high-dose statin and ezetimibe. PCSK9 inhibitors therapy was initiated by an FH specialist if LDL-C>70 mg/dl and the number of patients attending FH specialist doubled after the implementation of this pathway [7]. In the "SWEDEHEART" study, 82% of patients achieved LDL-C < 1.8 mmol/L at one year in comparison to 54% in 2014 before the implementation of the protocol. The number of patients achieving a target LDL-C < 1.4 mmol/L increased to 58% in 2019 [3,7]. In the Danish population study, 40.7% of patients achieved LDL-C levels < 1.8mmol/L and 92.3% of patients were on mild to intensive lipid-lowering therapy at six-month follow-up. The percentage of patients in this group on intensive lipid-lowering therapy was 8.1% at six months. There was a small decline in the percentage of patients who achieved the target LDL-C < 1.8 mmol/L to 39% at 12 months despite the fact that 32.4% of patients were on intensive lipid-lowering therapy by then. The percentage of patients on no lipid therapy also increased from 80.3% at six months to 86.7% at 12 months follow-up. Patients in Austria on combination therapy of statin and ezetimibe with LDL-C > 100 mg/dL two months following ACS were referred to an endocrinologist for PCSK9 inhibitors initiation. Patients in this study group with Dutch Lipid Clinic Network Score (DLCN) > 5 had a genetic screening for FH and 8% tested for FH [3,13]. Patients who achieved a reduction in LDL-C levels by 40% or LDL-C<70 mg/dl had PCSK9 inhibitors prescription renewed. In the Euroaspire V study, 51% of Spanish patients have LDL-C level >70 mg/dL (>1.8 mmol/L) a year following ACS and a key factor for this poor achievement of target LDL-C was a difference in the target level general practitioner and cardiologists. Patients in this study were followed up in a virtual clinic and 90% of patients had an LDL-C test prior to clinic appointments and combination therapy of high-intensity statins and ezetimibe was commenced in patients with LDL-C > 70 mg/dL. Patients on low to moderate statin doses had their doses increased if their LDL-C >70 mg/dL. These patients were referred to the hospital lipid clinic if LDL-C remained > 70 mg/dL for consideration of PCSK9 inhibitors. This significantly improved lipid control in patients and the percentage of patients achieving LDL-C < 70 mg/dL reached 64% in 2018 vs 30% in 2015 [3,13,14].

The French committee of cardiologists' consensus guideline for managing dyslipidaemia in patients with ACS was first implemented in 2017 at Besançon Hospital’s cardiology department and enrolled 270 patients between February 2017 and September 2018 [15,16]. Statin-naïve patients with LDL-C > 100 mg/dL received ezetimibe and it was also prescribed to patients on moderate-intensity statins with baseline LDL-C>70 mg/dL and patients on high-intensity statins with baseline LDL-C > 55 mg/dL. Around 95% of patients were discharged on high-intensity statins alone and 65% of patients were discharged on combination therapy of ezetimibe and statin and 68% of patients achieved a reduction of > 50% in their LDL-C values two months following ACS [13,14,17]. The first follow-up meeting in this study was organized within four to six weeks with either a cardiologist or a GP [14]. The Rivoli Hospital in Italy established a separate protocol to manage dyslipidaemia in patients with ACS who were discharged to GP for further management of dyslipidaemia. The general practitioners would receive a discharge summary for patients with clear LDL-C target value and if they were not achieved, they were referred to a lipid clinic for consideration of PCSK9 inhibitor therapy [2,17]. As a result of this initiative, patients were discharged on a combination therapy of statin and ezetimibe resulting in significant LDL-C improvement at 12 months following ACS [3,18]. The Lithuanian cardiology association extracted data from 1714 patients with early onset ischaemic heart disease below the age of 50 and these patients got screening for familial hypercholesterolaemia [19].

The Chinese Medical Doctors Association consensus guidelines state that targeting LDL-C levels < 1.8 - < 1.4 mmol/L for patients with ACS and LDL-C < 1.0 mmol/L is recommended in patients who suffer a second cardiac event within two years of the previous event. Based on the results from "The IMPROVE-IT" study, the target LDL-C < 1.8 mmol/L and < 1.4 mmol/L was achieved with high-intensity simvastatin monotherapy and combination therapy of high-dose simvastatin and ezetimibe respectively. The ODYSSEY Outcomes study showed that the LDL-C level in ACS patients treated with alirocumab was reduced to 1.38 mmol/L [20,21]. The Dyslipidaemia International Study II (DYSIS II) based on data from 10,661 patients from 18 countries demonstrated a significant increase in the percentage of patients followed up after ACS from 65.2% to 95.6%; 93.4% of patients were on statin monotherapy only. This study also highlighted that female gender, smoking, and high BMI had a negative correlation with target LDL-C level and type 2 diabetes mellitus, chronic kidney disease and high-intensity statin therapy had a positive correlation with target LDL-C level achievement [3,22]. The Dyslipidaemia International Study (DYSIS) II in Singapore enrolled 126 patients from 325 following ACS. The LDL-C level was 113.5 ± 43.9 mg/dL at admission and only 35% of patients achieved the target of less than LDL-C <70mg/dl at the four-month follow-up. As a result, a combination therapy of statin and ezetimibe or high-intensity statin therapy was initiated for these patients. The Dyslipidaemia International Study II (DYSIS II) in China enrolled 1103 patient were enrolled from 28 hospitals. About 20% of patients admitted with ACS were already established on statin therapy prior to an event and 41.2% of patients achieved LDL-C < 1.8mmol/L at the six-month follow-up following the cardiac event. The mean LDL‐C value at the six-month follow‐up was 2.1 ± 0.8 mmol/L; for patients on LLT and non-LLT, the LDL-C level was 2.2 ± 0.8 mmol/L and 2.1 ± 0.8 mmol/L, respectively. Most patients were on statin monotherapy and only 7.7 % of patients received a combination of statins and ezetimibe [23]. Another multinational study from Russia, Saudi Arabia, Colombia, Taiwan, Thailand, Malaysia, Indonesia, Singapore and Hong Kong demonstrated that only 47.1% of patients achieved target LDL-C levels < 70 mg /dL and based on the LDL-C values available from follow-up of these patients, the mean LDL-C was 77mg/dL. Of 922 patients, 776 were on high-dose statins; 81.8% of patients from this group of 776 patients achieved LDL-C < 100 mg/dL; 51.2% of patients achieved LDL-C levels < 70 mg/dL. Among 194 patients on low/moderate statin dose, 74.2% of patients achieved LDL-C < 100 mg/dL and 33% of patients achieved LDL-C < 70 mg/dL [24].

"The ACS patient pathway project", a multinational project in seven European countries enrolled 2775 patients (940 acute and 1835 follow up) demonstrated that 91% of patients had their lipid tested during the acute phase. The percentage of patients who had lipid tests during admission was 83% in the United Kingdom (UK) patients compared to 97% of patients in Italy [21]. Around 73% of patients had their lipid levels tested either on admission or the first day of admission whereas 16% of patients had their lipid levels checked about three or more days after admission. From this group, 93% of patients were on LLT and among them, 56% vs 37% were statin-naive. About 33% of patients in France were receiving a combination of high-intensity statin ± ezetimibe vs 88% in the UK. Similarly, a low to moderate intensity statin ± ezetimibe was prescribed to 5% of patients in the UK and 51% of patients in France. The follow-up plans were arranged for 79.4% of patients only, with the highest follow-up in Italy and Netherlands with 93% and 94% respectively. Only 64% of patients and their general practitioners received letters from their cardiologists on lipid therapy optimization and target lipid levels. On their first follow-up visit, only 22.4% of patients had LDL-C levels < 70 mg/dL and 77.6% of patients had LDL-C levels > 70 mg/dL. Only 10% of patients achieved LDL-C levels < 55 mg/dL on their first follow-up visit which increased to 16% on the third follow-up visit with the highest number of patients achieving this goal being from France [25]. About 21% of patients did not have any hospital follow-up arranged in this study and at the first follow-up visit, only 22.4% had LDL-C < 70 mg/dL, 37.8% had LDL-C between 70-99 mg/dL, and 40% had values > 100 mg/dL.

The Cooperative National Registry of Acute Coronary care, Guideline Adherence and Clinical Events “CONCORDANCE” is an Australian prospective registry of ACS patients from 43 different Australian Hospitals from 2009 to 2018 [24]. The study enrolled 4243 patients with ACS who got discharged from hospitals and lipid measurements were recorded for 2671 patients at the six and 12-month follow-up visits. The mean age for the study participant was 63 ± 12 years and 1951 participants from this group of 2671 were men. This study showed that about 45% of Australian patients were not on lipid-lowering therapy 12 months after suffering a major cardiac event. The study demonstrated that 1194 (45%) patients did not achieve the lipid target on 12-month follow-up. It also demonstrated that 334 (28%) patients who did not achieve the desired lipid target and 436 (30%) patients who did, were commenced on intensive lipid-lowering therapy. A total of 2077 patients including 876 (73%) of those who did not achieve and 1201 (81%) of those who achieved the recommended lipid targets were prescribed intensive lipid-lowering therapy at the time of discharge [26].

The comparison and study demographics for these studies are shown in Tables 1-2.

**Table 1:** Demographic characteristics of the review studies

Author	Country	Study	Setting (Hospital/GP)	Age/Age range	Patient No:
Alings M, et al. 2020 [3]	Netherland	PENELOPE Study algorithm	Hospital	NA	301
Gulizia MM, et al. 2020 [5]	Italy	POSTER study	Hospital	66 ± 11	5415
Descamps OS, et al. 2018 [6]	Belgium	The BEL-FaHST protocol (the Belgium Familial Hypercholesterolemia Strategy	Hospital	<65 years	NA
Kristensen MS, et al. 2020 [9]	Denmark	A Danish population-based cohort study	Hospital	67.8 ± 13.6	4377
García RV, et al. 2022 [13]	Spain	Follow-up virtual visits	Virtual follow-up by GP	67.3 ± 2.3	346
Ruiz-Bustillo S, et al. 2019 [14]	Sweden	A nurse-led lipid protocol study (SWEDEHEART registry)	Hospital	65-79	17214
Sabouret P, et al. 2022 [15]	France	IN-Hospital algorithm	Hospital	NA	270
Poh KK, et al. 2019 [19]	Singapore	The Dyslipidemia International Study (DYSIS) II	Hospital	64.8	325
Gong Y, et al. 2021 [20]	China	DYSIS II	28 hospital centres	61.7 ± 11.3	1103
Han Y, et al. 2020 [23]	Chinese	ODYSSEY Outcome study	Hospital	58.6	18924
Navar AM, et al. 2021 [24]	Multinational study	The Acute Coronary Syndrome Management (ACOSYM) registry	Hospital	59.9 ± 11.6	1567
Landmesser U, et al. 2020 [25]	Multinational	The ACS patient pathway project	Hospital	65.3 ± 12.5	2775
Brieger D, et al. 2019 [26]	Australia	CONCORDANCE	Hospital	63 ± 12	4243

**Table 2:** LDL-C levels during admission, follow-up and lipid-lowering therapy

COUNTRY	LDL-C post-MI	LDL-C 1^st^ follow-up	LDL-C 2^nd^ follow-up	Intervention on admission	Intervention on follow-up
Gulizia MM, et al. 2020 [5]	>1.8 mmol/L	< 1.8 mmol/L and < 1.4 mmol/L	1.38 mg/dl	Statin plus ezetimibe	PCSK9i
Descamps os, et al. 2018 [6]	LDL-C level>190 mg/dL or 130 mg/dL	LDL-C>70 mg/dl	> 190 mg/dL	High-intensity statin (or with addition of ezetimibe if already on high-intensity statin	PCSK9i
Kristensen MS, et al. 2020 [9]	89.4% patients had LDL-C > 1.8 mmol/L	59.3% patients had LDL-C > 1.8 mmol/L and 41.7% had LDL-C < 1.8 mmol/L.	61% patients had LDL-C > 1.8 mmol/L and 39% had LDL-C < 1.8mmol/L.	Statin	High dose statin and ezetimibe
Sabouret P, et al. 2022 [11]	LDL-C>100 mg/dL	>100 mg/dL	C<70 mg/dL	High statin therapy. Very high risk, high statin therapy and ezetimibe	High risk therapy and ezetimibe
Toplak H, et al. 2016 [12]	LDL-C>190 mg/dL or LDL-C>100 mg/dL if under statin	LDL-C >100 mg/dL	LDL-C decreased by 40% or LDL-C<70 mg/dl	High statin therapy and ezetimibe	PCSK9i
García RV, et al. 2022 [13]	>70 (>1.8 mmol/L)	>70 (>1.8 mmol/L)	LDL-C< 70 mg/dL	High intensity statin	High intensity statin ± ezetimibe ± PCSK9i
Ruiz-Bustillo S, et al. 2019 [14]	>1.8 mmol/L	LDL-C< 70 mg/dL	35% of the patients reach <1.4 mmo/L and 68% reach <1.8mmo/L and 82% achieve LDL-C < 1.8 mmol/L at 12 months	Statin and ezetimibe	PCSK9i
Han Y, et al. 2020 [23]	2.7 ± 0.9 mmol/L	Only 17% patients had LDL-C < 1.8 mmol/L	41.2% patients had LDL-C < 1.8 mmol/L	Statin only	Statin plus ezetimibe
Navar AM, et al. 2021 [24]	LDL-C > 70 mg/dL	47.1% patients achieved target LDL-C levels < 70 mg/dL	47.1% patients achieved target LDL-C levels < 70 mg/dL	Low or high-dose statin	Low or high-dose statin
Landmesser U, et al. 2020 [25]	91% patients had LDL-C > 70 mg/dl	83% patients had LDL-C > 70 mg/dl	68% patients had LDL-C > 70 mg/dl	Low or high-dose statin	High-dose statin and ezetimibe
Brieger D, et al. 2019 [26]	70 mg/dL	2.69 mg/dL in patients on statin and 2.64 mg/dL in patients not on statin	76% patients on LLT had LDL-C > 1.8 mmol/L and 74% patients not on LLT had LDL-C > 1.8 mmol/L	High or low-dose statin only	High or low-dose statin and ezetimibe

Issues with lipid management pathways following acute coronary syndrome

There are several issues with lipid management in patients who suffer acute myocardial infarction. The above lipid management initiatives highlighted several key issues including a lack of follow-up arrangements for patients at the time of discharge, delay in follow-up appointments, lack of timely optimization of lipid-lowering therapy, and lack of standard and unified lipid pathways. Patients are discharged to primary care in quite a few countries on discharge and there is a lack of clear communication between primary care and secondary care cardiologists. Primary care physicians are not provided with target lipid values for patients which hinders lipid therapy optimization. Medication intolerance was another issue that contributed to the sub-optimal lipid control to minimize future CVD risk. There is also variation between various guidelines, which is perhaps another reason resulting in different target LDL-C levels for patients depending on their geographical location. Another major issue identified in the ACS patient pathway project was that most patients despite being followed up in secondary care were not having repeat lipid profiles checked in secondary care, which contributed to sub-optimal lipid management putting them at future CVD risk. For example, 17% of patients in the UK did not have their lipid profile tested during the acute phase and during the follow-up phase, the percentage of patients who had their lipid profile tested dropped to 68%. A major concern raised in this study was that a significant percentage of patients had LDL-C levels > 100 mg/dL not only during admission but also at the follow-up stage, clearly pointing to a sub-optimal approach. Another issue highlighted by these studies was that most patients were discharged on sub-optimal therapies as only two-thirds received high-dose statins and a very small number of patients received combination therapy. This was perhaps partly due to pressures on hospitals for a quick turnover. A very small percentage of patients were commenced on PCSK9 inhibitors on their first follow-up suggesting that there is still much more work to be done. The ODYSSEY OUTCOMES study demonstrated that very high-risk patients who get treated with alirocumab have significantly lower cardiovascular risk and quite a significant number of these patients are eligible for PCSK9 inhibitors therapy on their first presentation. However, the above studies showed that cardiologists often disregard inappropriate pharmacological therapies for lipid management and do not optimize therapies in a timely manner.

Can cardiac rehabilitation integrated lipid management be the solution?

Jones (2022) performed a retrospective, post-pathway clinical audit at a local cardiac rehabilitation unit in Wales by incorporating a lipid management pathway into the cardiac rehabilitation program and the findings from this retrospective audit showed a significant improvement in lipid-lowering therapy optimization [27]. The data was collected for all patients referred to the cardiac rehabilitation programs between 1 June 2020 and 1 December 2020 following acute coronary syndrome. A total of 54 patients were identified to meet the criteria with a mean age of 69.4 years and the percentage of female and male participants was 38% and 62% respectively. The number of patients with NSTEMI and STEMI was 59% and 37%. Only 37.5% of female participants achieved target LDL-C levels compared to 61.5% of male participants before the new pathway integration into a cardiac rehabilitation program. The pathway was based on checking lipid profiles in patients attending rehabilitation programs at six weeks instead of three months recommended by NICE guidelines, hence these patients were identified earlier allowing timely intervention to reduce their future CVD risk. These include dietary modification, physical activity and medicine optimization by initiating combination therapy such as high-intensity statins and ezetimibe and onward referral. Female participants were more likely to achieve >50% reduction in LDL-C levels than male participants on the new cardiac rehab integrated pathway; however, male participants were twice more likely to achieve the target LDL-C levels < 1.4 mmol/L. About 70% of STEMI patients achieved a 50% reduction in their LDL-C levels compared to 47% of NSTEMI patients. The percentage of patients achieving the NICE-recommended 40% reduction in LDL-C levels increased by 48% after the implementation of the new cardiac rehab integrated lipid management pathway which is likely due to early follow-up in patients and timely medical optimization. The ESC guidelines also recommend a combination therapy of high-intensity statin and ezetimibe within four to six weeks following ACS if LDL-C target levels are not met and further optimization by initiating PCSK9 inhibitors in further four to six weeks if target LDL-C levels are not met. The ODYSSEY OUTCOMES and FOURIER studies demonstrated that PCSK9i therapy combined with potent lipid powering therapy resulted in a 15% relative reduction in the primary outcome and a 15% reduction in mortality from all causes after a median follow-up of 2.8 years. Similar findings were observed in The FOURIER study that included 27,564 patients with ASCVD and LDL-C ≥ 70 mg/dL receiving statin therapy and had a 15% reduction in the primary outcome following initiation of PCSK9 inhibitor therapy [28,29]. Cardiac rehabilitation provides an ideal opportunity for this in our opinion. This provides a unique opportunity and is worth exploring with further research to assess the effectiveness of cardiac rehab-integrated lipid management [27]. This was a retrospective study with a small sample size which are its two potential limitations, however, this is an area worth exploring given the fact that cardiologists often miss patients during the early rehabilitation phase to optimize their medical therapy. This is likely the time when patients are more self-conscious and receptive to any medical advice and this perhaps provides a window of opportunity and further research including randomized controlled trials might be useful.

Limitations

There are several limitations to this review. These include lack of limited data available online for most studies, sparsity of such studies from the developing world, lack of uniformity in the approach to the problem of dyslipidaemia in patients following acute myocardial infarction, and lack of uniformity in approaches in the management of dyslipidaemia. Finally, several studies did not provide sufficient information for all the variables thus limiting our ability to compare more studies. This was unlikely to change the overall findings but perhaps may have added some more information and could have increased the number of included articles in the study.

## Conclusions

Patients following acute coronary syndrome are at high risk for future events and sub-standard lipid management remains a major concern in these patients, putting at them higher risk. This concern is valid for both developing and developed countries. It is important to optimize lipid management for patients at risk of cardiac events, particularly those with a prior ACS event. Patients on optimal lipid-lowering therapy are at significantly lower risk to suffer from future cardiac events. There is considerable variation in follow-up for patients following acute cardiac events due to a lack of a uniform pathway in managing their dyslipidaemia in a timely manner as patients often get delayed secondary care appointments or get discharged to primary care and a short-lived window of opportunity to optimize their medical therapy is perhaps lost. Cardiac rehabilitation integrated lipid management might be an ideal solution to this problem as this will provide an early opportunity to identify those at risk and timely intervention can then be offered to them thus reducing their future CVD risk. 
